# Rare seizure presentation in 3-year-old male: Case of focal epilepsy associated with squatting and running

**DOI:** 10.1177/2050313X251364080

**Published:** 2025-07-31

**Authors:** Zayan Musa, Abhijnya Gowder, Herlinda Bergman, Roopa Viraraghavan

**Affiliations:** 1 Department of Medicine, University of California, Riverside, CA, USA

**Keywords:** pediatric seizures, focal epilepsy, breath-holding spells, periventricular leukomalacia, video-EEG, antiepileptic drugs, pediatric neurology

## Abstract

We report the case of a 3-year-old male initially observed to have breath-holding spells, squatting, and spontaneous crying who later developed symptoms of atypical seizures suggestive of focal epilepsy. Electroencephalogram confirmed focal seizures, while MRI revealed periventricular leukomalacia. Antiepileptic drugs were imperative to resolving the seizure symptoms, although complications arose with certain side effects. This case highlights the importance of identifying different characteristic seizure presentations and distinguishing breath-holding spells from atypical seizures through a timely electroencephalogram and neuroimaging to make an accurate diagnosis. In addition, we discuss the intricacy of diagnosing atypical seizures and the different ways in which they can present. Help the reader direct their attention to a valuable case report about childhood epilepsy and the uncommon symptoms associated. Highlighting the importance of identifying different characteristic seizure presentations and distinguishing breath-holding spells from atypical seizures through timely electroencephalogram and neuroimaging to make an accurate diagnosis.

## Introduction

Seizures in young children can present in a variety of ways, often leading to diagnostic challenges. The differential diagnosis for seizure-like activity includes both neurological and non-neurological conditions, such as breath-holding spells (BHSs), which are a benign condition seen in children. BHSs are relatively common in infants and toddlers, with a prevalence of up to 4.6% in otherwise healthy children.^1^ These spells are characterized by a brief cessation of breathing along with facial pallor or cyanosis in response to distress. Along with a temporary loss of consciousness, however, they can also present with stiffness or loss of bladder control, which are similar to presentations of atypical seizures.^2^ Distinguishing between these and seizures is critical, as management strategies and prognostic implications vary widely. When breath-holding is accompanied by other seizure-like features, as seen in this case, a comprehensive neurological evaluation, including an electroencephalogram (EEG), is essential.^3^

Focal epilepsy in children can present with many clinical manifestations, including motor symptoms, gaze deviation, and autonomic features. Diagnosis is confirmed through EEG studies, which can identify the seizure focus and guide treatment decisions.^4^ Etiologies of focal epilepsy in children include metabolic disorders, genetic factors, and structural causes such as periventricular leukomalacia (PVL). PVL is caused by inflammatory or ischemic cerebral damage to white matter adjacent to the lateral ventricles of the brain. Although commonly known to cause neurological impairment in premature infants, PVL can also be seen in full-term infants.^5^ MRI findings may include periventricular white matter lesions, ventricular dilation, and cortical atrophy, as seen in this case.

This case report highlights an atypical presentation of focal epilepsy in a 3-year-old male, initially suspected to be a benign BHS with unique squatting behaviors and episodic crying. This case emphasizes the need for careful clinical evaluation and diagnostic workup, including EEG monitoring, to differentiate between these two conditions and guide appropriate treatment. Parental consent was given to analyze and publish this case.

## Case

The 3-year-old male presented to the Emergency Department (ED) with a new onset of unusual behavioral episodes. The mother reported that the child’s episodes included breath-holding, facial redness, squatting, spontaneous crying, and limb stiffness, with each episode lasting ~1 and 10–15 min apart. During these episodes, he did not lose consciousness or exhibit typical seizure signs such as eye deviation or tonic-clonic movements. There was no reported history of head trauma preceding the onset of symptoms, and the patient displayed no evidence of post-ictal confusion and instead displayed a burst of energy, often running around the house or crying.

During his ED visit, the patient appeared alert with no distress, but the frequency and nature of his episodes raised concern. Differential diagnoses included both BHSs and a true seizure disorder. Due to the observation of limb shaking and stiffness, an underlying seizure disorder was suspected, and a neurology consult was initiated. Video-EEG monitoring was recommended to differentiate between these possible diagnoses; however, this was not completed due to the patient exhibiting additional symptoms before the appointment date, which led them back to the Emergency Room (ER).

Approximately 1 month later, the patient presented again to the ED due to evolving behavioral episodes, which now lasted 20–30 s. New symptoms included facial twitching, upward gaze deviation, self-biting, and urinary incontinence post-episode in addition to previous symptoms of breath-holding and crying. These new symptoms prompted further exploration of an alternative diagnosis to BHSs.

Neurological workup was done, and the video-EEG showed focal epilepsy localized to the left temporal region of the brain, documenting 22 electro-clinical seizures. This confirmed the diagnosis of epilepsy, distinct from the initial suspicion of BHSs.

## Hospital course and management

After diagnosis, seizure management was initiated with antiepileptic drugs (AEDs). The patient was started on Levetiracetam at a dose of 20 mg/kg/day BID, with a loading dose of 60 mg/kg IV. After the loading dose, the patient experienced a cluster of seizures, which were controlled with Lorazepam. The next day, another seizure cluster necessitated a more aggressive regimen, including additional Levetiracetam, Lorazepam, Midazolam, and a second Levetiracetam loading dose. These interventions successfully halted the seizures.

Due to frequent seizures and concern for status epilepticus, the patient was then transferred to the Pediatric Intensive Care Unit (PICU) for closer monitoring. Video-EEG continued, and Lacosamide was added, significantly reducing seizure frequency. By discharge, the seizures were infrequent, with only mild shaking and stiffness noted during these episodes.

During this admission, a brain MRI revealed multiple T2 FLAIR hyperintensities in both occipital lobes, which were interpreted as findings consistent with PVL. The absence of masses confirmed that no significant structural damage was contributing to the seizures.

One week later, the patient was discharged in stable condition, having remained seizure-free and adherent to his treatment. He was discharged on a regimen of Levetiracetam, Lacosamide, and Topiramate for seizure prevention, with Diazepam as rescue therapy for breakthrough seizures.

Two weeks after discharge, the patient presented at his neurology follow-up with complaints of excessive tantrums (yelling, hitting) after starting Levetiracetam, though he showed improvement in other aspects of recovery, such as improved appetite. Mild strabismus was also noted. The family is now awaiting follow-up with an external neurology clinic for further steps.

## Discussion

This case highlights an atypical seizure presentation, specifically focal epilepsy, which can be mistaken for benign behavioral conditions in children. Diagnosing atypical seizures is challenging, but recognizing their diverse symptoms and distinguishing them from common atypical seizure types can aid in making an accurate diagnosis, helping with specific treatment.

While focal seizures may present with behavioral symptoms and staring episodes, they often occur fewer times per day and are usually accompanied by motor dysfunction.^6^ Their presentation can be highly variable, sometimes mimicking benign conditions such as BHSs. BHSs typically resolve spontaneously and do not present with features such as loss of consciousness, tonic-clonic activity, abnormal eye movements, or postictal confusion.^7^ The presence of these symptoms should prompt further neurological evaluation for potential seizure activity. This patient’s spontaneous crying is also a documented manifestation of focal seizures, although rare. Notably, cases of focal seizures involving the left temporal lobe—such as in this patient—have presented with crying as a primary symptom.^8^

Absence seizures are another common atypical pediatric seizure type alongside focal seizures, making differentiation between the two essential due to their distinct clinical features. Absence seizures are generalized and typically lack motor involvement.^6^ Approximately 60% of affected patients also experience neurological symptoms, including mood disturbances, memory deficits, and attention difficulties.^9^ This condition is frequently associated with learning and behavior impairments and is characterized by recurrent staring episodes, sometimes up to 30 times/day. Another common cause of epilepsy is traumatic brain injury, observed in many pediatric cases.^10^ Although the mechanism is not fully understood, modern antiepileptic medications have not been shown to be a permanent solution for late-onset seizures and warrant further investigation for prevention and treatment.

Imaging confirmed a diagnosis of focal epilepsy in this patient, affecting sensation, eye movements, and motor function.^4^ Early medical intervention and imaging were crucial to his care, as video-EEG successfully pinpointed the seizure focus in the left temporal lobe—a common site for epileptic seizures and thus an important factor in characterizing the disorder.^11^ Typical medical management with AEDs was implemented, in this case, with lacosamide and levetiracetam, which successfully improved his symptoms.^4^ Recent studies have also shown that gene therapy and altering gene expression can target focal epilepsies and serve as a novel way to treat this condition.^12^ Targeting specific genes not only allows for more precision, but it also serves as a more permanent solution to treat epileptic seizures. In addition, modern fornicotomy, which uses tools such as laser ablation and high-frequency ultrasound ablation to disrupt the fornix to treat temporal lobe seizures, has been recently proven to show good outcomes compared to previous surgical fornicotomies.^13^

In addition to video-based EEG, the patient underwent brain MRI to evaluate for potential structural abnormalities. Imaging findings were interpreted as consistent with PVL, likely related to complications from preterm birth. MRI remains the gold standard for diagnosing PVL. However, it is important to consider leukodystrophies in the differential, as they can present with similar white matter abnormalities on imaging. PVL is typically associated with prematurity and perinatal hypoxic-ischemic events, whereas leukodystrophies are inherited disorders often characterized by early-onset visual impairment, progressive cognitive decline, and spastic paralysis.^14^ Although evidence indicates that PVL raises the risk of seizures, more investigation is required to comprehend the connection.^5^

## Conclusion

This case revealed an instance of focal epilepsy that presented as a BHS, highlighting the importance of distinguishing these serious neurological disorders from benign conditions. The use of EEG and neuroimaging was instrumental in the accurate diagnosis of this child’s condition, and early intervention was key in successfully getting control of the seizures. Due to this, the prognosis for this patient remains optimistic with regular follow-ups, careful observation of his condition, and appropriate medication adjustment.
